# Communication Skills Training: Using Evidence to Develop Programs That Improve Patient Outcomes

**Published:** 2013-05-01

**Authors:** Lisa Kennedy Sheldon

**Affiliations:** From University of Massachusetts Boston, College of Nursing and Health Sciences, Boston, Massachusetts

It is well accepted that patient-centered communication is a cornerstone of quality oncology care (Epstein & Street, 2007; Institute of Medicine, 2007). Patient-provider communication involves complex processes, especially in oncology care. Training in effective communication has been proposed for over 3 decades, and many communication skills training (CST) programs have been developed to improve these skills in oncology health-care providers. However, the lack of consistency in topics, methods, analyses, and outcome variables has led to the development of training programs based more on expert opinion and less evidence-based approaches. Barth and Lannen’s systematic review in *Annals of Oncology* (2011) uses meta-analytic approaches to examine the effects of CST interventions on provider and patient outcomes. The purpose of this paper is to discuss the implications for CST programs for health-care providers in oncology care within the context of Barth and Lannen’s article.

## Background

Effective CST begins during the process of formal education for health-care providers. In oncology, providers may receive their initial education during training in their respective professions such as medicine, nursing, social work, pastoral care, and mental health care. However, further training may be needed after graduation, when new providers apply these skills independently in clinical practice. It is during this time postgraduation that health-care providers develop a professional communication style. Some acquired communication behaviors may be beneficial to patient outcomes, such as the use of empathic responses, while others, such as blocking communication, may be detrimental to the patient assessment or potentially harmful to patients. Therefore, postgraduate training programs need to be implemented after formal education when independent professional communication styles are being developed and formalized.

In addition, while self-efficacy may improve over the course of time, empathic responsiveness may actually decline over years of clinical practice. "Booster" CST programs may be particularly useful in redeveloping empathic skills and enhancing professional communication in experienced clinicians.

## Communication Skills

In their article, Barth and Lannen (2011) note that the main purpose of CST programs in oncology is to increase empathic behaviors and the clarity of patient-provider communication as well as to address more challenging communication scenarios. Communication is conceptualized as a set of basic skills that can be taught to providers. de Haes and Bensing, leaders in the field of patient-provider communication, have developed a new model to direct future research in health-care provider and patient communication (de Haes & Bensing, 2009). Their model links elements of communication processes to specific goals and then patient-related outcomes. It describes six functions of communication: (1) fostering the relationship, (2) gathering information, (3) providing information, (4) making decisions, (5) enabling disease- and treatment-related behavior, and (6) responding to emotions. Patient outcomes are categorized as immediate, intermediate, and long-term endpoints and include trust, health, self-efficacy, and satisfaction with decisions. Linking the functions of communication with specific provider behaviors is necessary to achieve the desired patient-related outcomes.

Barth and Lannen (2011) noted the heterogeneity of the CST programs, a problem from many standpoints when analyzing effectiveness. Communication skills training programs were variable in length, lasting from a few hours to several days. These programs may or may not include a booster or consolidation session after the initial training. In this meta-analysis, Barth and Lannen (2011) only reviewed studies that included CST interventions that lasted at least 6 hours and included role-play activities. Evaluation of provider behaviors after CST was done primarily by audio or video recording communication of providers role-playing with either real or "simulated" patients (actors). The results of the meta-analysis indicated that longer programs had larger effect sizes, with more benefit if a booster session was included as part of the program.

Postgraduate CST programs have been developed to meet the needs of individual professions and sometimes the needs of patients. Specific provider skills have included breaking bad news (Paul, Clinton-McHarg, Sanson-Fisher, Douglas, & Webb, 2009), dealing with emotional issues (Razavi et al., 2002), and communicating at the end of life (Alexander, Ketz, Sloane, & Tulsky, 2006; Fellowes, Wilkinson & Moore, 2004; Sheldon, 2005; Gyselis, Richardson, & Higginson, 2005). These programs often target one profession, such as doctors (Merckaert et al., 2008), but sentinel studies have also included multiple professions (Maguire, Booth, Elliot, & Jones, 1996) and tested programs in interprofessional communication (Krimshtein et al., 2011). However, in their investigation, Barth and Lannen (2011) found few studies that explored patient outcomes other than satisfaction with provider communication and adherence to prescribed treatment recommendations.

## Newer Goals

Communication is a dyadic process: providers communicate with patients/families and vice versa. Despite the dyadic nature of this communication, the participants in CST programs are most often health-care providers, e.g., educational interventions to teach providers how to communicate with patients. Teaching patients how to communicate with health-care providers is a newer goal of CST interventions. Patient preferences for communication with their oncology care providers are needed to inform and guide the development of CST programs. In one study, researchers assessed the effectiveness of patient self-report of symptom and quality-of-life (SQOL) issues and found that these tools increase patient-provider communication regarding SQOL (Berry et al., 2011) concerns. In a newer study, the same researchers are assessing the impact of a patient teaching tool to help patients communicate with oncology providers about their symptoms and concerns. Ultimately, it may be more efficient and effective to teach communication skills to both patients and health-care providers.

Establishing outcomes for communication is essential to designing CST programs that meet patient needs. These programs provide training in communication skills that both facilitate patient disclosure and develop responsiveness in providers to patient questions and concerns. While expert opinion and experiential wisdom provide valuable insight into necessary communication skills, evidence regarding the effectiveness of specific skills is needed to develop and refine CST programs. For example, the National Comprehensive Cancer Network (NCCN) recommends that providers assess patient distress by facilitating discussion and assessment for potentially treatable issues such as distress and depression (NCCN, 2012). Additionally, by 2015, screening and treatment/referral for psychosocial distress will be a required standard of clinical management for cancer programs accredited by the American College of Surgeons (ACOS, 2011). Most oncology providers are not formally trained in psychological services and may need CST programs to learn effective communication skills to assess psychosocial concerns, respond appropriately, and identify treatable conditions—all skills that will facilitate timely referral and treatment.

## Future Research

More research is needed to establish the ideal length of training sessions and programs, the need for booster sessions, and the trajectory for maintenance of acquired skills. Barth and Lannen recommend courses of at least 3 days’ length with booster session/consolidation workshops at a later date. They also recommend the use of role-playing to practice and evaluate communication skills. While longitudinal programs may offer more durable effects, there is currently no sufficient evidence to make this recommendation other than the experiential wisdom that communication skills can be refined and deepened over time. Certainly, experienced clinicians understand both the need for training and the ongoing development of these skills over years of practice. Given that the recommended CST programs occur after graduation, when providers are in clinical practice, it might be more efficient to conduct them at workplaces and in shorter segments to allow more clinicians to participate.

## Conclusion

Barth and Lannen (2011) acknowledge the urgent need to evaluate the effectiveness of CST programs on patient outcomes. They acknowledge a gap between programs and clinical impact that could be better addressed through the identification of specific communication outcomes and consistency in training. It is time to apply a theoretical framework, such as the model proposed by de Haes and Bensing, and current evidence from controlled trials and meta-analyses to guide the development of these programs and clearly link CST interventions to desired patient-related outcomes. In addition, providing postgraduate education to practicing clinicians may best occur at worksites in smaller segments. Training both providers and patients may be the most effective way to improve communication in all facets of oncology care. Finally, patient perspectives are needed to inform and develop CST programs and improve patient outcomes.
